# Glycogen synthase kinase‐3β activity plays a key role in the antitumor effect of nafamostat mesilate in pancreatic cancer cells

**DOI:** 10.1002/ags3.12025

**Published:** 2017-08-31

**Authors:** Koichiro Haruki, Hiroaki Shiba, Yohta Shimada, Yoshihiro Shirai, Ryota Iwase, Yuki Fujiwara, Tadashi Uwagawa, Toya Ohashi, Katsuhiko Yanaga

**Affiliations:** ^1^ Department of Surgery The Jikei University School of Medicine Tokyo Japan; ^2^ Division of Gene Therapy Research Center for Medical Science The Jikei University School of Medicine Tokyo Japan

**Keywords:** glycogen synthase kinase‐3β, nafamostat mesilate, nuclear factor‐kappa B, pancreatic cancer, protein phosphatase 2

## Abstract

Pancreatic cancer is often resistant to chemotherapy. We previously showed the efficacy of combination treatment using gemcitabine and nafamostat mesilate (FUT‐175) for patients with unresectable pancreatic cancer. However, the mechanisms that affect the sensitivity of FUT‐175 are not fully understood. The purpose of the present study was to clarify the mechanism of the sensitivity to FUT‐175, with a focus on the activity of glycogen synthase kinase‐3β (GSK‐3β). In vitro, we assessed sensitivity to FUT‐175 in human pancreatic cancer cell lines (PANC‐1 and MIAPaCa‐2) and difference of signaling in these cells by cell proliferation assay, Western blot analysis and microarray. Next, we assessed cell viability, apoptotic signal and nuclear factor‐kappa B (NF‐κB) activity in response to treatment with FUT‐175 alone and in combination with GSK‐3 inhibitor or protein phosphatase 2A (PP2A) by cell proliferation assay, Western blot analysis and enzyme‐linked immunosorbent assay. Phosphorylated GSK‐3β level was significantly higher in MIAPaCa‐2 (high sensitivity cell) than in PANC‐1 (low sensitivity cell). Cell viability and NF‐κB activity were significantly decreased by addition of GSK‐3 inhibitor to FUT‐175, and levels of cleaved caspase‐8 were increased by inhibition of GSK‐3. PP2A inhibitor increased the levels of phosphorylated GSK‐3β and sensitized both cell lines to FUT‐175 as measured by cell viability and apoptotic signal. The results indicate that GSK‐3β activity plays a key role in the antitumor effect of FUT‐175 in pancreatic cancer cells, and regulation of GSK‐3β by PP2A inhibition could be a novel therapeutic approach for pancreatic cancer.

## 1 INTRODUCTION

Pancreatic cancer is one of the most lethal and aggressive of all malignancies and the fourth leading cause of death in developed countries.^1^ Because of rapid tumor growth and high potential for distant metastasis at the time of diagnosis, one‐half of patients present with metastatic disease and approximately the other 30% with locally advanced unresectable disease.^2^ Multidisciplinary therapy has been applied to advanced pancreatic cancer, but the median overall survival remains unsatisfactory.

Recent studies have shown that nuclear factor‐kappa B (NF‐κB) plays an important role in regulation of cell apoptosis, inflammation, and oncogenesis,^3^, ^4^ and constitutive NF‐κB activation contributes to the aggressive behavior of pancreatic cancer.^5^ Chemotherapeutic agents and ionizing radiation activates NF‐κB, which leads to resistance against chemoradiation therapy.^6^, ^7^ Therefore, inhibition of NF‐κB activation in cancer cells may be one of the potential options to enhance the antitumor effect of chemoradiation therapy. We previously reported that nafamostat mesilate (FUT‐175), a serine protease inhibitor^8^ that is widely used in Japan for the treatment of pancreatitis and disseminated intravascular coagulation as well as an anticoagulant for hemodialysis, inhibited NF‐κB activation; it also induced caspase‐8‐mediated apoptosis of pancreatic cancer cells in vitro^9^ and in vivo.^10^ Moreover, FUT‐175 inhibited anticancer agents‐ or radiation therapy‐induced NF‐κB activation and enhanced antitumor effects in pancreatic,^11^, ^12^, ^13^ gallbladder,^14^ and gastric cancer.^15^ We also reported the usefulness of gemcitabine in combination with intra‐arterial FUT‐175 administration for unresectable pancreatic cancer patients in phases 1 and 2 clinical studies.^16^, ^17^ Other studies investigated new therapeutic options to sensitize pancreatic cancer cells to chemotherapeutic agent^18^ and several clinical trials of pancreatic cancer have already targeted molecular factors, either as monotherapy or in combination with gemcitabine.^19^ Our treatment strategy has targeted NF‐κB activation using FUT‐175 in pancreatic cancer cells. However, similar to other chemotherapeutic agents, the sensitivity to FUT‐175 was different in each cell line. To improve the therapeutic outcome of pancreatic cancer and to develop a novel treatment strategy, it is important to investigate the mechanisms that affect the sensitivity of FUT‐175.

The purpose of the present study was to clarify the mechanism of the sensitivity of FUT‐175 in order to improve therapeutic outcome of pancreatic cancer by combination therapy with FUT‐175.

## 2 MATERIALS AND METHODS

### 2.1 Reagents

FUT‐175 was kindly given to us by Torii Pharmaceutical Co., Ltd (Tokyo, Japan), which was dissolved in sterile distilled water (5 mg/mL) and stored at −20°C until use. Glycogen synthase kinase (GSK)‐3 Inhibitor IX, and cantharidic acid as protein phosphatase 2A (PP2A) inhibitor was purchased from Merck Millipore (Darmstadt, Germany), which were dissolved in dimethyl sulfoxide (10 mmol/L) and stored at 4°C and room temperature until use. Protease inhibitor cocktail and phosphatase inhibitor cocktail tablets were purchased from Roche Diagnostics (Indianapolis, IN, USA).

### 2.2 Cell culture

The human pancreatic cancer cell lines PANC‐1 and MIAPaCa‐2 were purchased from American Type Culture Collection (Rockville, MD, USA). PANC‐1 and MIAPaCa‐2 were maintained in Dulbecco's modified Eagle's medium (DMEM) containing 10% fetal bovine serum (FBS) (GIBCO BRL, Grand Island, NY, USA) and penicillin/streptomycin (GIBCO BRL). The cells were cultured at 37°C with 5% CO_2_.

### 2.3 Antibodies

Antibodies specific to phosphorylated AKT, phosphorylated GSK‐3β and cleaved caspase‐8 were obtained from Cell Signaling Technology (Beverly, MA, USA). Anti‐Dishevelled 2 antibody was obtained from Abcam (Cambridge, MA, USA) and Anti GSK‐3β was obtained from BD Biosciences (San Jose, CA, USA). Anti‐β‐actin antibody was purchased from Sigma Chemical (St Louis, MO, USA).

### 2.4 In vitro experimental treatment groups

PANC‐1 and MIAPaCa‐2 cells were treated with FUT‐175 (80 μg/mL) (FUT‐175 group), GSK‐3 Inhibitor or PP2A inhibitor (10 μmol/L) (inhibitor group), each inhibitor (10 μmol/L) and FUT‐175 (80 μg/mL) (combination group), and without any treatment (control group) for an appropriate time in each analysis. In the combination group, PANC‐1 and MIAPaCa‐2 cells were treated with each inhibitor for 24 hours before treatments. These samples were used for quantitative analysis of microarray, polymerase chain reaction (PCR), NF‐κB activity, cell proliferation assay and Western blot analysis. All experiments were independently carried out in triplicate. Concentration of both GSK‐3 Inhibitor and PP2A inhibitor were determined according to previous studies.^20^, ^21^

### 2.5 Microarray assay

Total RNA was extracted using SV Total RNA Isolation System (Promega, Madison, WI, USA). Experiments were carried out by a contract research service at Dragon Genomics Center (Takara Bio, Mie, Japan). Cy3‐labeled cRNA was prepared with 500 ng RNA using a Quick Amp labeling Kit, one‐color (Agilent, Santa Clara, CA, USA) according to the manufacturer's protocol. Expressed genes were screened using SurePrint G3 Human GE 8×60 K v2 Microarray (Agilent) according to the manufacturer's instructions. Gene expression was compared using a log ratio, which was defined as log_2_ (PANC‐1/MIAPaCa‐2).

### 2.6 Quantitative analysis of NF‐κB activity

Concentrations of NF‐κB p65 in the nuclear extracts were measured. Nuclear extracts from in vitro experiments were prepared using a nuclear extract kit (Active Motif, Carlsbad, CA, USA) according to the manufacturer's protocol. The nuclear extracts were assayed using an ELISA kit (TransAM™ NF‐κB; Active Motif) to detect and quantify the NF‐κB activity according to the manufacturer's instructions. Briefly, 20 μg of the nuclear extract protein was incubated for 1 hour at 25°C in microwells coated with an oligonucleotide containing an NF‐κB p65 or p50‐binding consensus sequence. Next, the wells were incubated with rabbit anti‐NF‐κB p65 antibodies (1:1000 dilution) for 1 hour at 25°C, followed by incubation with peroxidase‐conjugated goat anti‐rabbit IgG (1:1000 dilution) for 1 hour at 25°C. Peroxidase activity was visualized by the tetramethylbenzidine reaction, and the optical density was measured at 450 nm.

### 2.7 Cell proliferation assay

PANC‐1 and MIAPaCa‐2 cells were seeded into 96‐well plates (1×10^4^ cells in each well). PANC‐1 and MIAPaCa‐2 were incubated with each treatment for 24 hours. Cell proliferation was measured with a Cell Titer 96 Aquenous One Solution Cell Proliferation Assay (Promega) following the manufacturer's instructions.

### 2.8 Western blot analysis

Lysate protein was extracted from whole cells (5×10^6^ cells for each) after each treatment for 24 hours. This protocol for Western blot analysis was described in a previous study.^22^ After incubating the blots in each primary antibody overnight, the membranes were incubated with the peroxidase‐labeled secondary antibody (Histofine; Nichirei, Tokyo, Japan) for 2 hours and detected by using the ImmunoStar LD^®^ chemiluminescence reagent (WAKO Chemical, Tokyo, Japan). Protein bands were detected using a Chemi Doc XRS+ system (Bio‐Rad, Hercules, CA, USA).

### 2.9 Reverse transcription polymerase chain reaction

Total RNA was extracted using the SV Total RNA Isolation System (Promega), and reverse transcription (RT) was carried out using a Transcriptor First Strand cDNA synthesis Kit (Roche Applied Science, Mannheim, Germany) according to the manufacturer's instructions. Primer sequences for the genes were as follows:

5′‐CACTCCCGCTACATCACCAC‐3′ (forward),

5′‐ACACCGTCTCCATCCACATC‐3′ (reverse) for protein phosphatase 2, regulatory subunit B, alpha (PPP2R2A) and

5′‐GGCAAGCGGAGAAAAGACGA‐3′ (forward),

5′‐AAAACACTGGACCCACCAGA‐3′ (reverse) for protein phosphatase 2, regulatory subunit B, beta (PPP2R2B) and

5′‐GACGCAGGATGGAAGACAGA‐3′ (forward),

5′‐CATTGTCAGAGGGGCAGAGT‐3′ (reverse) for protein phosphatase 2, regulatory subunit B, gamma (PPP2R2C) and

5′‐CGCTCTCTGCTCCTCCTGTT‐3′ (forward),

5′‐CATCGCCCCACTTGATTTTG‐3′ (reverse) for glyceraldehyde‐3‐phosphate dehydrogenase (GAPDH). RT‐PCR products were visualized by ethidium bromide‐stained agarose gels.

### 2.10 Statistical analysis

Data were expressed as mean±SD. Non‐paired *t‐*test (two‐tailed) was used for statistical studies. All *P*‐values were considered statistically significant when the associated probability was <.05.

## 3 RESULTS

### 3.1 Cell growth inhibition of PANC‐1 and MIAPaCa‐2 by FUT‐175

**Figure 1:**
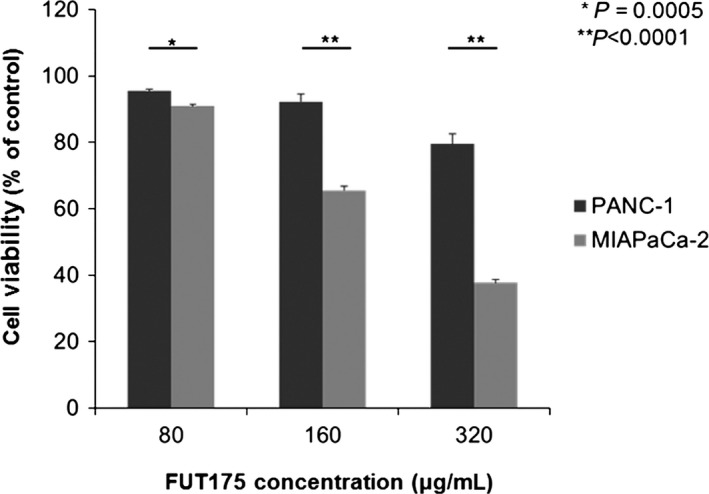
Cell viability of MIAPaCa‐2 was significantly lower than that in PANC‐1 at each concentration of FUT‐175 (80 μg/mL, *P*<.0001; 160 μg/mL, *P*<.0001; 320 μg/mL, *P*<.0001)

We previously reported that FUT‐175 had an antitumor effect against pancreatic cancer.^10^ To clarify the difference of sensitivity to FUT‐175 in human pancreatic cancer cells, we examined cell viability after treatment with FUT‐175 by the MTT assay using PANC‐1 and MIAPaCa‐2. By the cell proliferation assay, cell viability of MIAPaCa‐2 was significantly lower than that of PANC‐1 at each concentration of FUT‐175 (80 μg/mL, 90.8% vs 95.5%, *P*<.0001; 160 μg/mL, 65.5% vs 92.3%, *P*<.0001; 320 μg/mL, 37.7% vs 79.5%, *P*<.0001; Figure 1).

### 3.2 Assessment of expression changes of genes by FUT‐175

To investigate the change in mRNA expression of PANC‐1 and MIAPaCa‐2, we carried out microarray analyses. As to GSK signaling, AKT2 and DVL‐1 were greater in PANC‐1 than in MIAPaCa‐2. PPP2R2A was lower in PANC‐1, whereas the expressions of PPP2R2B and PPP2R2C were greater in PANC‐1 than in MIAPaCa‐2 (Table 1).

**Table 1:** Comparison of gene expression around GSK signaling in PANC‐1 and MIAPaCa‐2 cells

Accession no. mRNA	Description	Log_2_ ratio^a^	Scale signal	
			MIAPaCa‐2	PANC‐1
NM_005163	*AKT1*	−0.06	12 948.3	12 404.4
NM_001626	*AKT2*	3.48	807.4	9013.9
NM_004421	*DVL1*	1.20	1462.9	3350.5
NM_004422	*DVL2*	0.36	5427.9	6948.5
NM_002093	*GSK3B*	−0.86	3642.5	2008.7
NM_002717	*PPP2R2A*	−2.01	9801.1	2432.4
NM_004576	*PPP2R2B*	3.02	69.8	567.7
NM_181876	*PPP2R2C*	2.49	396.4	2228.0

a Log ratio is defined as log_2_ (PANC‐1/MIAPaCa‐2).

GSK, glycogen synthase kinase.

### 3.3 Assessment of AKT signal in PANC‐1 and MIAPaCa‐2 treated by FUT‐175

**Figure 2:**
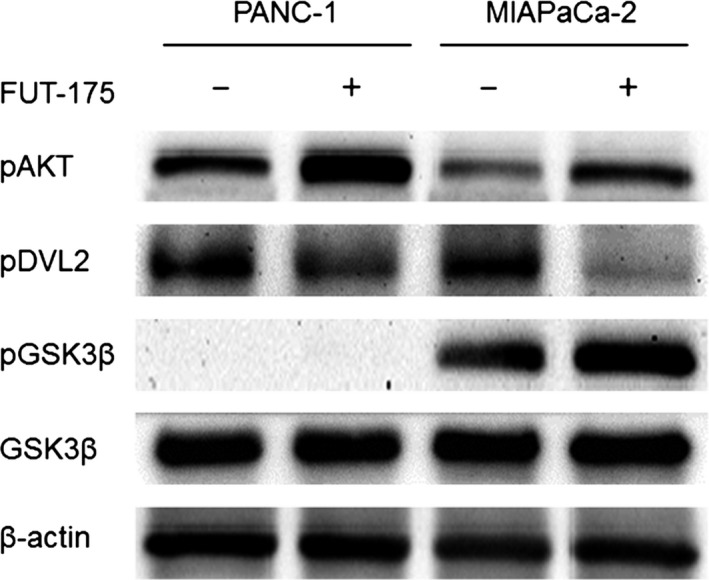
Levels of phosphorylated AKT and phosphorylated DVL in PANC‐1 were greater than in MIAPaCa‐2. However, the level of phosphorylated GSK‐3β was significantly greater in MIAPaCa‐2 than in PANC‐1. Phosphorylated AKT was increased by FUT‐175 in both cell lines, whereas phosphorylated DVL was decreased by FUT‐175 in both cell lines

Based on the results of the microarray, we evaluated the AKT signal, the Wnt signal and the activity of GSK‐3 by Western blot analysis. Although the levels of phosphorylated AKT and phosphorylated DVL in PANC‐1 were greater than in MIAPaCa‐2, levels of phosphorylated GSK‐3β in PANC‐1 were not detected. Phosphorylated AKT was increased by FUT‐175 in both cell lines, whereas phosphorylated DVL was decreased by FUT‐175 in both cell lines (Figure 2).

### 3.4 Enhancement of the antitumor effect of FUT‐175 by inhibition of GSK‐3 activity

**Figure 3:**
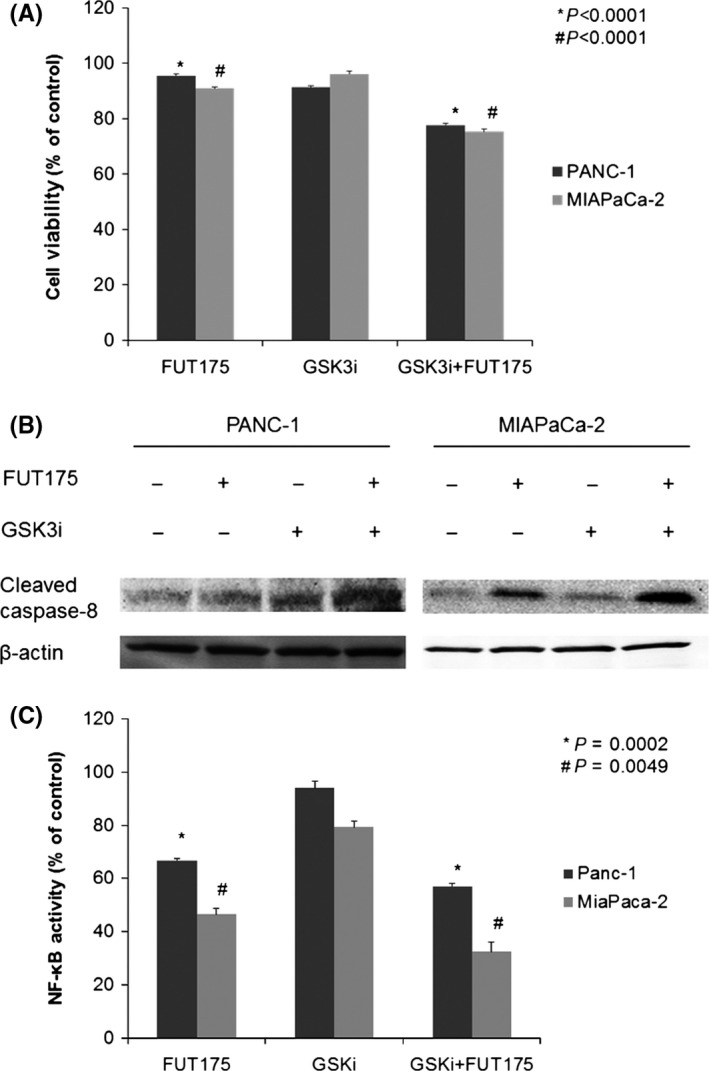
(A) Cell viability of both pancreatic cancer cell lines was significantly decreased by addition of glycogen synthase kinase (GSK)‐3 inhibitor to FUT‐175 (PANC‐1, *P*<.0001; MIAPaCa‐2, *P*<.0001). (B) In Western blot analysis, levels of cleaved caspase‐8 of both pancreatic cancer cell lines were increased by the addition of GSK‐3 inhibitor to FUT‐175, which were greater than those of FUT‐175 alone. (C) Concentrations of nuclear factor‐kappa B (NF‐κB) p65 in the nuclear extracts of PANC‐1 and MIAPaCa‐2 cells were significantly decreased by the addition of a GSK‐3 inhibitor to FUT‐175, which were lower than those of FUT‐175 alone (PANC‐1, *P*=.0002; MIAPaCa‐2, *P*=.0049) (C)

To evaluate whether inhibition of GSK‐3 activity affected the antitumor effect of FUT‐175, we examined cell viability and apoptotic signal in the pancreatic cancer cell lines treated by FUT‐175 with GSK‐3 inhibitor. In the cell proliferation assay, the viability of both pancreatic cancer cell lines was significantly decreased by the addition of GSK‐3 inhibitor to FUT‐175 (PANC‐1, *P*<.0001; MIAPaCa‐2, *P*<.0001, Figure 3A). In Western blot analysis, the levels of cleaved caspase‐8 of both pancreatic cancer cell lines were increased by the addition of GSK‐3 inhibitor to FUT‐175, which were greater than those of FUT‐175 alone (Figure 3B).

Furthermore, we evaluated whether GSK‐3 inhibition affected NF‐κB activity. NF‐κB is typically a heterodimer that consists of p65 and p50 proteins. To assess NF‐κB activity, we measured concentrations of p65 in the nuclear extracts by ELISA. Concentrations of NF‐κB p65 in the nuclear extracts of PANC‐1 and MIAPaCa‐2 cells were significantly decreased by the addition of the GSK‐3 inhibitor to FUT‐175, which were lower than those of FUT‐175 alone (PANC‐1, *P*=.0002; MIAPaCa‐2, *P*=.0049, Figure 3C).

### 3.5 PP2A regulated GSK‐3 activity and inhibition of PP2A improved sensitivity to FUT‐175

**Figure 4:**
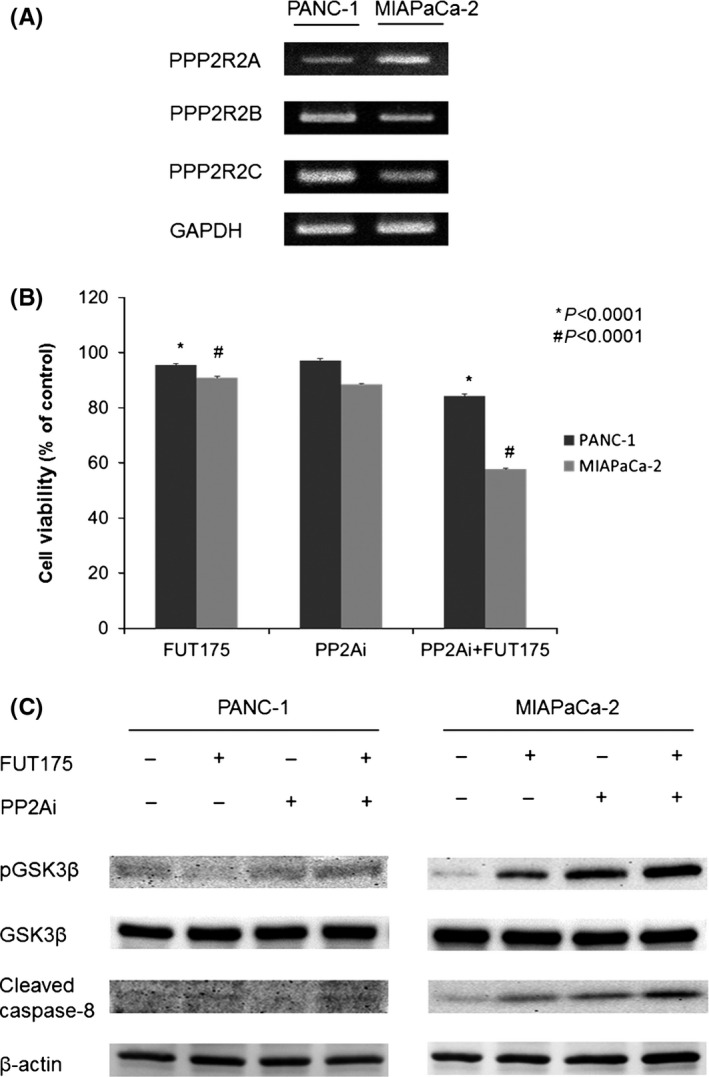
(A) In reverse transcription‐polymerase chain reaction (RT‐PCR), expression of PPP2R2A was lower in PANC‐1, whereas expressions of PPP2R2B and PPP2R2C were greater in PANC‐1 than in MIAPaCa‐2. (B) Cell viability of both pancreatic cancer cell lines were significantly decreased by addition of PP2A inhibitor to FUT‐175 (PANC‐1, *P*<.0001; MIAPaCa‐2, *P*<.0001). (C) In Western blot analysis, levels of phosphorylated GSK‐3β were increased in the PP2A group compared to those of the control group. By combination of FUT‐175 and PP2A inhibitor, levels of phosphorylated glycogen synthase kinase (GSK)‐3β were greater than those of FUT‐175 alone. Levels of cleaved caspase‐8 of both pancreatic cancer cell lines were increased in line with the presence of phosphorylated GSK‐3β

To investigate the detailed molecular mechanism for the difference of GSK‐3 activity between PANC‐1 and MIAPaCa‐2, we examined the expression of PP2A regulatory subunits by RT‐PCR. PP2A plays essential roles in many fundamental cellular functions, including cell proliferation, migration and survival and regulates several oncogenic signaling cascades.^23^ PP2A was reported to directly regulate GSK‐3 activity through dephosphorylation of GSK‐3β regardless of upstream signals such as pAKT or Wnt.^24^, ^25^ As a result of microarray analyses, in RT‐PCR, expression of PPP2R2A was lower in PANC‐1, whereas expressions of PPP2R2B and PPP2R2C were greater in PANC‐1 than in MIAPaCa‐2 (Figure 4A). It is suggested that GSK‐3 was activated by PP2A in PANC‐1 through dephosphorylation, which explains the low sensitivity to FUT‐175.

To evaluate whether inhibition of PP2A affects the antitumor effect of FUT‐175 and phosphorylated GSK‐3β level, we examined cell viability, GSK‐3 activity and apoptotic signal in the pancreatic cancer cell lines treated by FUT‐175 with PP2A inhibitor. In the cell proliferation assay, the viability of both pancreatic cancer cell lines was significantly decreased by the addition of PP2A inhibitor to FUT‐175 (PANC‐1, *P*<.0001; MIAPaCa‐2, *P*<.0001, Figure 4B). In Western blot analysis, levels of phosphorylated GSK‐3β were increased in the PP2A group compared to those in the control group. By a combination of FUT‐175 and PP2A inhibitor, levels of phosphorylated GSK‐3β were greater than those of FUT‐175 alone. Levels of cleaved caspase‐8 of both pancreatic cancer cell lines were increased in line with the presence of phosphorylated GSK‐3β (Figure 4C).

## 4 DISCUSSION

Pancreatic cancer is highly resistant to current chemotherapeutic agents, and the therapeutic outcomes remain unsatisfactory. Therefore, new therapeutic approaches are needed to improve the outcome of pancreatic cancer. Our clinical trials provided both acceptable overall survival and other clinical advantages such as a reduction in opioid analgesic consumption and a healthy weight gain in patients with unresectable pancreatic cancer.^17^ Moreover, FUT‐175 has more potential effects, reducing recurrence through inhibition of cell adhesion or invasion and increasing sensitivity to anoikis.^26^ We have now started a phase 2 trial of gemcitabine and TS‐1 in combination with regional arterial infusion of FUT‐175 for patients with unresectable pancreatic cancer and have experienced a conversion curative resection case of primarily unresectable pancreatic cancer after this regimen.^27^ Above all, although FUT‐175 is widely used for patients with severe disease, its safety is a strong point of this combination therapy. Furthermore, identifying biomarkers for target treatment is important to improve the therapeutic effect.

**Figure 5:**
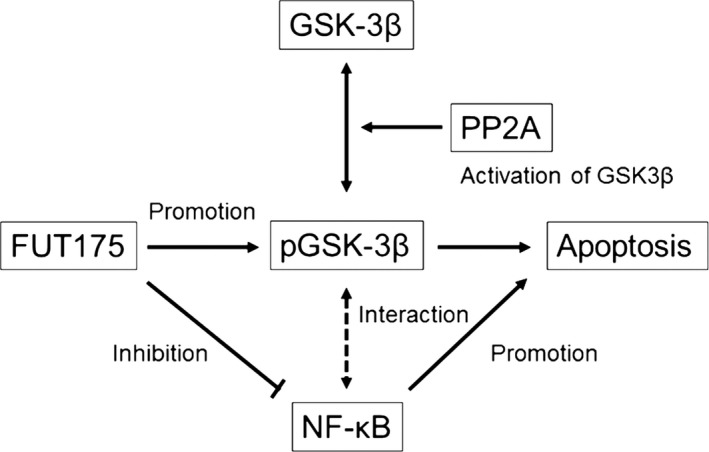
Schematic diagram of the role of glycogen synthase kinase (GSK)‐3β in the signaling pathways of the antitumor effect of nafamostat mesilate. The antitumor effect of nafamostat mesilate is in proportion to the level of phosphorylated GSK‐3β (inactive form of GSK‐3β) and nafamostat mesilate may enhance its antitumor effect by increasing phosphorylated GSK‐3β. PP2A inhibitor increases phosphorylated GSK‐3β as a result of inhibition of activation of GSK‐3β by PP2A, and enhances the antitumor effect of nafamostat mesilate. There is an interaction between nuclear factor‐kappa B (NF‐κB) and GSK‐3β

GSK‐3β is a protein kinase involved in the regulation of cell cycle, transcription, proliferation, differentiation, and apoptosis. Dysregulation of GSK‐3β has been implicated in tumor genesis and cancer progression.^28^ Recent studies have reported GSK‐3β as a strong and clinically meaningful prognostic biomarker in pancreatic cancer.^29^ Regulation of GSK‐3β has been a viable target in the treatment of pancreatic cancer as a result of its involvement in tumor development and chemoresistance.^30^ Moreover, GSK‐3β positively regulates NF‐κB and inactivation of GSK‐3β inhibits NF‐κB activity in pancreatic cancer cells.^31^, ^32^ In the current study, we demonstrated that the level of phosphorylated GSK‐3β, an inactive form of GSK‐3β, correlated with the antitumor effect of FUT‐175, and that regulation of GSK‐3β activity enhanced caspase‐8‐mediated apoptosis and the inhibitory effect of NF‐κB by FUT‐175. We regulated GSK‐3β by using GSK‐inhibitor and PP2A inhibitor. PP2A is an important and ubiquitously expressed serine phosphatase and regulates function by dephosphorylating many critical cellular molecules such as Akt, p53, c‐Myc, and β‐catenin.^23^ Structurally, it is multifarious as it is composed of catalytic, scaffold and regulatory subunits. A few studies suggest a relationship between pancreatic cancer and PP2A. PP2A promotes pancreatic cancer development by sustaining hyperactivity of multiple oncogenic signaling pathways, including AKT, ERK, and Wnt,^33^ and inhibition of PP2A repressed the invasive ability of pancreatic cancer cells.^21^ These studies also provide a basis for exploring PP2A as a diagnostic or therapeutic target in pancreatic cancer. In the current study, we first demonstrated in pancreatic cancer cells that inhibition of PP2A contributed to the regulation of GSK‐3β through dephosphorylation of GSK‐3β. This result suggested that regulation of PP2A could play a key role in the treatment of pancreatic cancer not only by FUT‐175 but also by other therapeutic drugs. Figure 5 shows a schematic diagram of the role of GSK‐3β in the signaling pathways of the antitumor effect of nafamostat mesilate.

Consequently, GSK‐3β activity may be important for the therapeutic outcome of our clinical trial using FUT‐175. Clinical application of this study was to identify the level of GSK‐3β or PP2A in a biopsy sample or in an endoscopic ultrasound‐guided fine‐needle aspiration sample before giving FUT‐175 to patients with pancreatic cancer. From these results, we can make clinical decisions regarding the treatment regimen for borderline and unresectable pancreatic cancer, which will be useful for identifying patients with poorer prognosis because of chemoresistance. To improve the therapeutic outcome of pancreatic cancer, further investigation to clarify the relationship between GSK‐3 and PP2A is needed.

## DISCLOSURE

Conflict of Interest: Authors declare no conflicts of interest for this article.
